# Immune Imbalance in Nasal Polyps of Caucasian Chronic Rhinosinusitis Patients Is Associated with a Downregulation of E-Selectin

**DOI:** 10.1155/2014/959854

**Published:** 2014-06-03

**Authors:** Michael Könnecke, Robert Böscke, Anja Waldmann, Karl-Ludwig Bruchhage, Robert Linke, Ralph Pries, Barbara Wollenberg

**Affiliations:** Department of Otorhinolaryngology, University Medical Center Schleswig-Holstein, Campus Luebeck, Ratzeburger Allee 160, Luebeck, Germany

## Abstract

Chronic rhinosinusitis with nasal polyps (CRSwNP) in Caucasians is a chronic Th2 inflammatory disease of the nasal and paranasal mucosa and the recruitment of leukocytes to the site of inflammation is poorly understood. We studied mRNA and protein expression profiles of adhesion molecules in nasal polyp and associated inferior turbinate tissues using molecular, biochemical, and immunohistological methods. Analysis showed a strongly decreased E-selectin expression in nasal polyps with a significant difference between eosinophil and neutrophil counts in nasal polyps and balanced counts in inferior turbinates. E-selectin expression is known to be downregulated in a Th2 milieu and has an essential role in immunosurveillance by locally activating neutrophil arrest and migratory function. A downregulation of E-selectin may come along with an immune imbalance in Caucasian nasal polyps due to a significant inhibition of neutrophil recruitment. Therefore, we suggest that an upregulation of E-selectin and the associated influx of neutrophils may play a significant role in the resolution of inflammation as well as for the pathophysiology of nasal polyps of Caucasian chronic rhinosinusitis patients.

## 1. Introduction

Chronic rhinosinusitis (CRS) is a significant health problem [1] and recent data have illustrated that CRS affects about 5–15% of the population in Europe and the USA [2]. Chronic rhinosinusitis with nasal polyps (CRSwNP) is considered a subgroup of chronic rhinosinusitis (CRS), a chronic inflammatory condition of the nasal and paranasal sinuses. Clinical symptoms include chronic nasal congestion, nasal discharge, facial pressure, and hyposmia. CRSwNP is characterized by grape-like structures in the upper nasal cavity that originate from within the ostiomeatal complex.

Typical histological features of nasal polyps are a dense inflammatory infiltrate, loose fibrous connective tissue with substantial tissue edema, and a thickened basement membrane covered mostly by respiratory pseudostratified epithelium [2]. It has been proposed that the inflammatory reaction in nasal polyps is initiated by a reaction of multiple cell types (epithelial cells, lymphocytes, eosinophils, fibroblasts, and mast cells) to bacterial superantigens secreted by* Staphylococcus aureus* [3]. The reaction to* Staphylococcus aureus* superantigen potentially results in Th2-skewing with significantly elevated levels of interleukin- (IL-) 5, IL-13, eosinophil cationic protein (ECP), and eotaxin, generation of local polyclonal IgE, degranulation of mast cells, Treg inhibition, alteration of eicosanoid metabolism, and promotion of eosinophil survival. For a subgroup of polyps in Asian patients, the inflammation pattern was found to be distinctly different from the western population, showing Th1/Th17-skewed and the so-called “key cytokine negative” cytokine profiles [4].

The recruitment and activation of eosinophils in CRS requires the local expression of eosinophil-attracting chemokines by the epithelium and other cell types, as well as priming and survival promoting effects of cytokines such as GM-CSF and IL-5 [2]. The expression of adhesion molecules by the endothelium promotes the recruitment of leukocytes into the site of inflammation in a multistep process involving leukocyte adhesion, rolling along the surface of activated endothelial cells, and transendothelial migration [5]. Adhesion molecules involved in the recruitment of leukocytes include L-selectin, P-selectin, and E-selectin, which interact with P-selectin glycoprotein ligand 1 (PSGL1), as well as other glycosylated ligands, for example, intercellular adhesion molecule 1 (ICAM1) and vascular cell-adhesion molecule 1 (VCAM1) [6]. L-selectin is expressed by most leukocytes, whereas E-selectin, P-selectin, ICAM1, and VCAM1 are expressed by inflamed endothelial cells [6]. PSGL1 has a dominant role as a ligand for all three selectins and is expressed on almost all leukocytes as well as endothelial cells [7].

The known role of adhesion molecules for the pathophysiology of inflammatory diseases led us to investigate the mRNA und protein expression profiles of adhesion molecules in nasal polyps of chronic rhinosinusitis patients.

## 2. Materials and Methods

### 2.1. Patient Specimens

All patients were treated surgically at the Department of Otorhinolaryngology, University Hospital of Schleswig-Holstein, Campus Lübeck. The study was approved by the local ethics committee of the University of Schleswig-Holstein, Campus Lübeck, and conducted in accordance with the ethical principles for medical research formulated in the WMA Declaration of Helsinki. All participants have given their written informed consent. Nasal polyp tissue and associated inferior turbinate tissue, as internal control, were harvested from 34 Caucasian patients (28 males and 6 females, mean age 49.32 ± 14.10) who underwent functional endoscopic sinus surgery (FESS) or septoplasty with reduction of the inferior turbinates. All patients had a history of sinus-related inflammation of more than 3 months with a mean duration of 1.7 years (±0.8 years) and did not respond to conservative therapy. The Lund-Mackay scoring system (0–24) [8] was used to grade the radiographic occupancy of the sinus disease. All patients suffered from bilateral polyposis, and the mean Lund-Mackay score of the patients was 16.1 (±4.6). The endoscopic findings were classified according to the Lund-Kennedy scoring system (0–14) [9] and the mean score of the patients was 10.5 (±1.6). Quality of life was evaluated by the Sino-Nasal Outcome Test-20, German Adapted Version [10, 11], and a mean value of 34.2 (±13.2) was observed from the patient evaluations. Fresh tissue samples were flash frozen in liquid nitrogen immediately after resection, stored at −80°C before mRNA and protein extraction, and additionally embedded for cryostat sections. For microarray analysis nasal polyp tissue and inferior turbinate tissue of seven patients (6 males and 1 female, mean age 50.28 ± 9.35) were analyzed representatively. All patients had been free of steroid medication for at least 4 weeks and had no history of atopy, bronchial asthma, or salicylate intolerance.

### 2.2. Microarrays

Frozen tissues were shipped on dry ice to Miltenyi Biotec (Bergisch Gladbach, Germany) for microarray analysis. mRNAs were isolated using standard mRNA extraction protocols (Trizol) and were quality-checked via the Agilent 2100 Bioanalyzer platform (Agilent Technologies). The RNA integrity number (RIN) value was calculated and RNA with a RIN number >6 was used [12]. Agilent Whole Human Genome Microarrays (4 × 44 K) were performed following the manufacturer's protocols. The Rosetta Resolver gene expression data analysis system (Rosetta Biosoftware) was used to compare two single intensity profiles in a ratio experiment (inferior turbinate versus nasal polyp).

### 2.3. Quantitative Real-Time PCR

The microarray results were confirmed by quantitative real-time PCR (qPCR) using TaqMan gene expression assays (Applied Biosystems, Foster City, CA, USA, Table 1). The transcriptional activity of studied genes was analyzed using a LightCycler 1.5 (Roche). 1 *μ*g of each mRNA sample was synthesized to cDNA using the RevertAid First Strand cDNA Synthesis Kit (Fermentas, St. Leon-Rot, Germany) according to the manufacturer's instructions. qPCR reaction mixture consisted of 10 *μ*L TaqMan gene expression master mix (Applied Biosystems, Foster City, CA, USA), 1 *μ*L TaqMan gene expression assay (Applied Biosystems, Foster City, CA, USA), and 3 ng of cDNA. The thermal cycling conditions for qPCR were as follows: 50°C for 2 min for UDG incubation and 95°C for 10 min for AmpliTaq Gold, UP enzyme activation, 50 cycles at 95°C for 15 s, and 60°C for 1 min. To ensure that the endogenous control was stable expressed in nasal polyps and inferior turbinates we tested different endogenous controls such as *β*-actin, GAPDH, or HPRT1. After this, all selected gene mRNA levels in patients were measured and normalized to *β*-actin as reference gene. The 2^−ΔΔCt^ method [13] was used to analyze the qPCR data.

### 2.4. Western Hybridisation

Tissues were homogenized and proteins were isolated using 2 mL RIPA-buffer (1% Igepal, 0.5% Na-deoxycholate, 0.1% SDS, and 98.4% PBS) containing 60 *μ*L aprotinin, 20 *μ*L PMSF, 20 *μ*L pepstatin A, 20 *μ*L leupeptin, and 20 *μ*L PIC (phosphatase inhibitor cocktail) for each tissue. Protein concentrations were determined using Bradford protein assay with bovine serum albumin as standard. 90 *μ*g proteins for each tissue were diluted 1 : 1 in 2x protein buffer, heated to 65°C for 15 minutes, and separated by SDS-polyacrylamide gel electrophoresis (SDS-PAGE) using a 10% Mini-PROTEAN TGX Precast Gel (Bio-Rad, Hercules, CA, USA) containing internal standards (Kaleidoscope Precision Plus Protein Standards, Bio-Rad, Hercules, CA, USA). Proteins were electrophoretically transferred on a nitrocellulose membrane (0.45 *μ*m, Bio-Rad, Hercules, CA, USA) using the Mini Trans-Blot Cell (Bio-Rad, Hercules, CA, USA). Blots were blocked in 20 mM Tris base and 137 mM sodium chloride (TBS) containing 5% milk powder and 0.1% Tween 20 (TBS-T), for 60 min at room temperature and incubated overnight at 4°C with mouse monoclonal antibody against E-selectin (CD62E, ab24723, Abcam PLC, Cambridge, MA, USA), diluted 1 : 500 in TBS-T containing 3% milk powder. Blots were rinsed with TBS-T 3 times and incubated for 60 minutes at room temperature with an anti-mouse HRP-linked antibody (number 7076, Cell Signaling, Danvers, MA, USA), diluted 1 : 2000 in TBS-T containing 3% milk powder. After TBS-T rinse, protein bands were detected on the Fusion FX7 (Vilber Lourmat, Torcy, France) using the ECL method (Amersham Biosciences, Buckinghamshire, UK). To control the equal loading of the wells, blots were stripped and immunostained as described above, using mouse monoclonal antibody against ACTB (number 3700, Cell Signaling, Danvers, MA, USA), diluted 1 : 1000 in TBS containing 3% milk powder and anti-mouse HRP-linked antibody (number 7076, Cell Signaling, Danvers, MA, USA), diluted 1 : 2000 in TBS-T containing 3% milk powder.

### 2.5. Immunohistochemistry

Frozen tissue sections were air dried and fixed in ice-cold (−20°) acetone for 10 min. Immunostaining was performed using the labeled streptavidin biotin (LSAB) method. Sections were incubated for 15 minutes with 3% H_2_O_2_, rinsed three times in TBS, and incubated overnight with mouse monoclonal antibody CD62E (1 : 50; ab81195, Abcam PLC, Cambridge, MA, USA) or mouse monoclonal antibody CD62P (1 : 5000; ab6632, Abcam PLC, Cambridge, MA, USA). After washing in TBS, sections were incubated with polylink biotinylated antibody for 20 minutes, followed by peroxidase labeled avidin-biotin-complex for 20 min. Visualization was achieved by a final incubation with 3-amino-9-ethylcarbazole-peroxidase substrate chromogen. Sections were counterstained with Mayer's hematoxylin and expression levels were evaluated using the following grading system: negative, 1+, 2+, and 3+. Immunostaining was assessed in blinded fashion by two independent observers.

Eosinophil and neutrophil counts were evaluated on frozen tissue sections using two specific antibodies. For eosinophil detection we used an antibody against the human eosinophil major basic protein (BZL002004, Biozol, Eching, Germany; 1 : 50) and for neutrophils we used an antibody against neutrophil elastase (GTX72042, GeneTex, Irvine, CA, USA; 1 : 50). The number of positive cells was counted in 10 parts of high-power fields (HPF) at 400x magnification and scored by two independent observers. Afterwards, numbers of eosinophils and neutrophils were correlated with levels of E-selectin.

### 2.6. Statistical Analysis

For statistical analysis and graphs, Prism software (GraphPad, San Diego, USA) was used. Experiments were performed in triplicates. Means and standard deviations were compared using Wilcoxon matched-pairs signed rank test. *P* values ≤ 0.05 were considered to be statistically significant.

## 3. Results

### 3.1. Decreased mRNA Levels of E-Selectin in Nasal Polyps of CRS Patients by Microarray Analysis

Nasal polyp tissues showed significantly (*P* ≤ 0.01) altered gene expression values for over 4,000 genes and a significant increase of Th2 cytokines (IL-4, IL-5, IL-10, and IL-13) compared to the inferior turbinate (data not shown).

**Figure 1:**
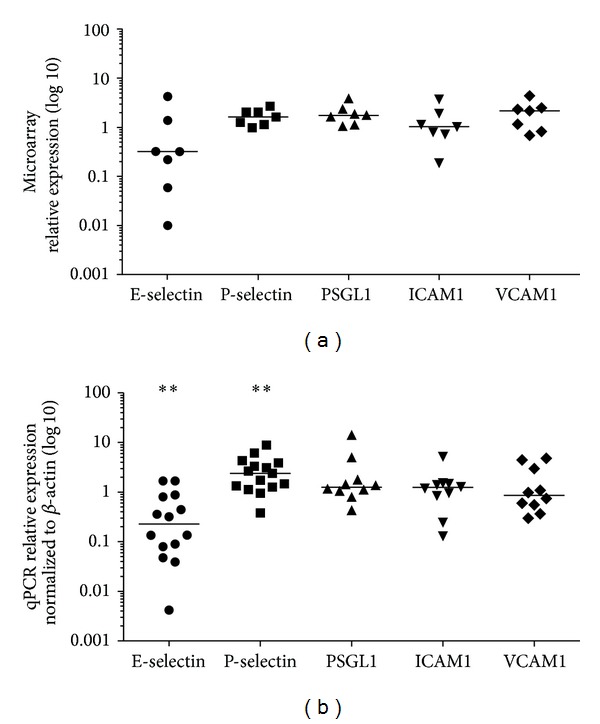
Scatter plots of adhesion molecule expression in nasal polyps compared to associated inferior turbinates using microarray and qPCR. Each single dot shows the relative expression of the target molecule in nasal polyps compared to associated inferior turbinates of one patient and median is indicated as* horizontal bar*. (a) Microarray analysis: nasal polyp tissues showed higher gene expression levels of the adhesion molecules P-selectin (1.62-fold, *n* = 7), PSGL1 (1.74-fold, *n* = 7), and VCAM1 (2.16-fold, *n* = 7) compared to associated inferior turbinates. ICAM1 was unregulated (1.03-fold, *n* = 7) and E-selectin was strongly downregulated in nasal polyps (0.32-fold, *n* = 7). (b) qPCR analysis: lower median expression of E-selectin (0.22-fold, *n* = 14) and higher median expression of P-selectin (2.37-fold, *n* = 15) were observed in nasal polyps. The median expression of PSGL1, ICAM1, and VCAM1 showed no significant difference between nasal polyps and inferior turbinates: PSGL1 1.24-fold (*n* = 10), ICAM1 1.24-fold (*n* = 10), and VCAM1 0.85-fold (*n* = 10). The mRNA levels of E-selectin in nasal polyps decreased significantly (*P* = 0.004) compared to the inferior turbinates and P-selectin increased significantly (*P* = 0.0025). PSGL1, ICAM1, and VCAM1 were expressed at similar levels.

Nasal polyp tissues showed higher gene expression levels of the adhesion molecules P-selectin (1.62-fold, *n* = 7), PSGL1 (1.74-fold, *n* = 7), and VCAM1 (2.16-fold, *n* = 7) compared to associated inferior turbinates (Figure 1(a)). ICAM1 was unregulated (1.03-fold, *n* = 7). Interestingly, E-selectin was strongly downregulated in nasal polyps (0.32-fold, *n* = 7).

### 3.2. Expression Levels Confirmed by Quantitative Real-Time PCR

**Figure 2:**
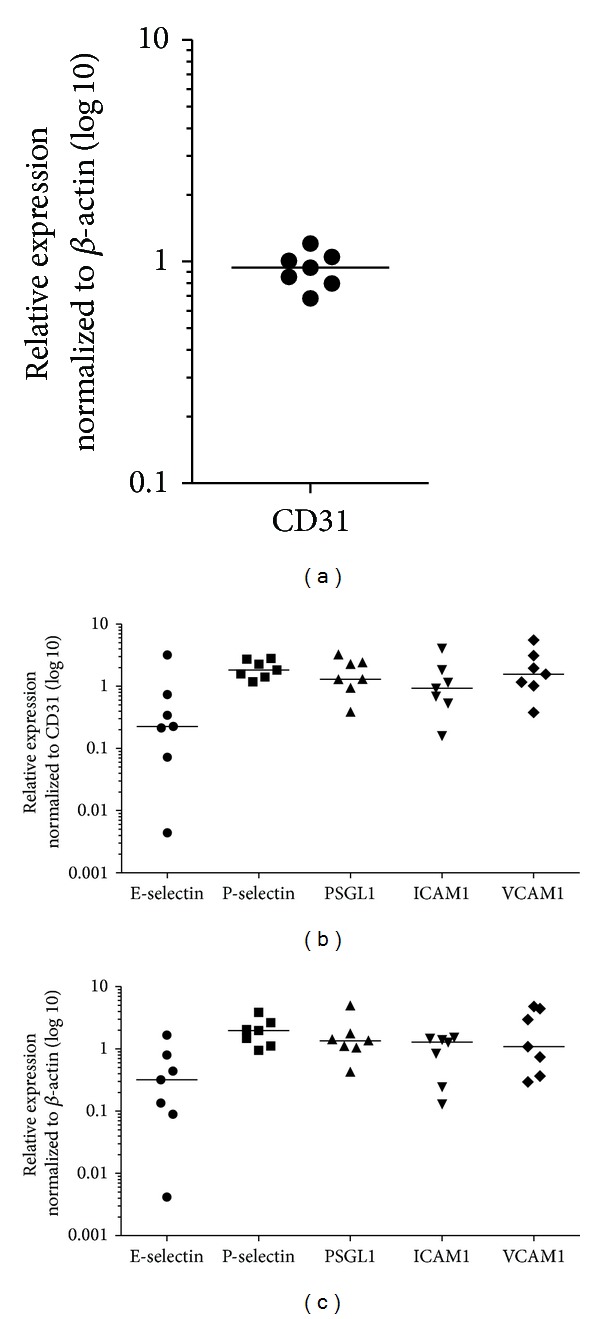
Scatter plots of CD31 and adhesion molecule expression in nasal polyps compared to associated inferior turbinates using qPCR. Each single dot shows the relative expression of the target molecule in nasal polyps compared to associated inferior turbinates of one patient and median is indicated as* horizontal bar*. (a) CD31 is not differentially expressed (0.93-fold, *n* = 7) between nasal polyps and inferior turbinates; (b) adhesion molecule expression in nasal polyps normalized to CD31. E-selectin (0.22-fold, *n* = 7), P-selectin (1.82-fold, *n* = 7), PSGL1 (1.29-fold, *n* = 7), ICAM1 (0.93-fold, *n* = 7), and VCAM1 (1.56-fold, *n* = 7). (c) Adhesion molecule expression in nasal polyps normalized to *β*-actin. E-selectin (0.31-fold, *n* = 7), P-selectin (1.97-fold, *n* = 7), PSGL1 (1.34-fold, *n* = 7), ICAM1 (1.28-fold, *n* = 7), and VCAM1 (1.08-fold, *n* = 7). CD31 normalized data differ insignificantly compared to the *β*-actin normalized data.

Median expression of E-selectin was decreased (0.22-fold, *n* = 14), while P-selectin expression was increased (2.37-fold, *n* = 15) in nasal polyps compared to associated inferior turbinates (Figure 1(b)). Expression levels of PSGL1, ICAM1, and VCAM1 did not significantly differ between nasal polyps and inferior turbinates (Figure 1). Median expressions were as follows: PSGL1 1.24-fold (*n* = 10), ICAM1 1.24-fold (*n* = 10), and VCAM1 0.85-fold (*n* = 10). Secondarily, we quantified the mRNA levels (Figure 1(b)). Adhesion molecules were variably expressed in nasal polyps and inferior turbinates. PSGL1, ICAM1, and VCAM1 were not significantly differently expressed. The mRNA level of E-selectin in nasal polyps decreased significantly compared to the inferior turbinates (*n* = 14, *P* = 0.004) and the mRNA level of P-selectin increased significantly (*n* = 14, *P* = 0.0025). Due to the fact that E-selectin, P-selectin, PSGL1, ICAM1, and VCAM1 are expressed by inflamed endothelial cells, we determined the expression of CD31, a specific endothelial cell marker, in nasal polyps and inferior turbinates of 7 patients. In fact CD31 is not differentially expressed (0.93-fold) between nasal polyps and inferior turbinates (Figure 2(a)). Additionally, we normalized our data to CD31 (Figure 2(b)) and the differences were insignificantly compared to the *β*-actin normalized data (Figure 2(c), Table 2).

### 3.3. Decreased Protein Levels of E-Selectin in Nasal Polyps of CRS Patients

**Figure 3:**
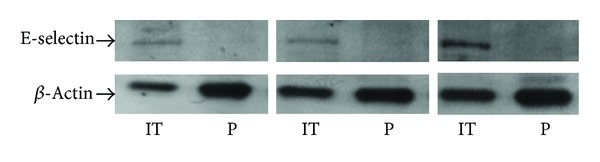
Western blotting exhibited lower E-selectin expression in nasal polyps (P) than in the inferior turbinate (IT) of chronic rhinosinusitis patients (*n* = 8).

Protein analysis by Western blotting showed strongly decreased E-selectin expression levels in nasal polyps compared to the inferior turbinates of chronic rhinosinusitis patients adapted to *β*-actin (Figure 3).

**Figure 4:**
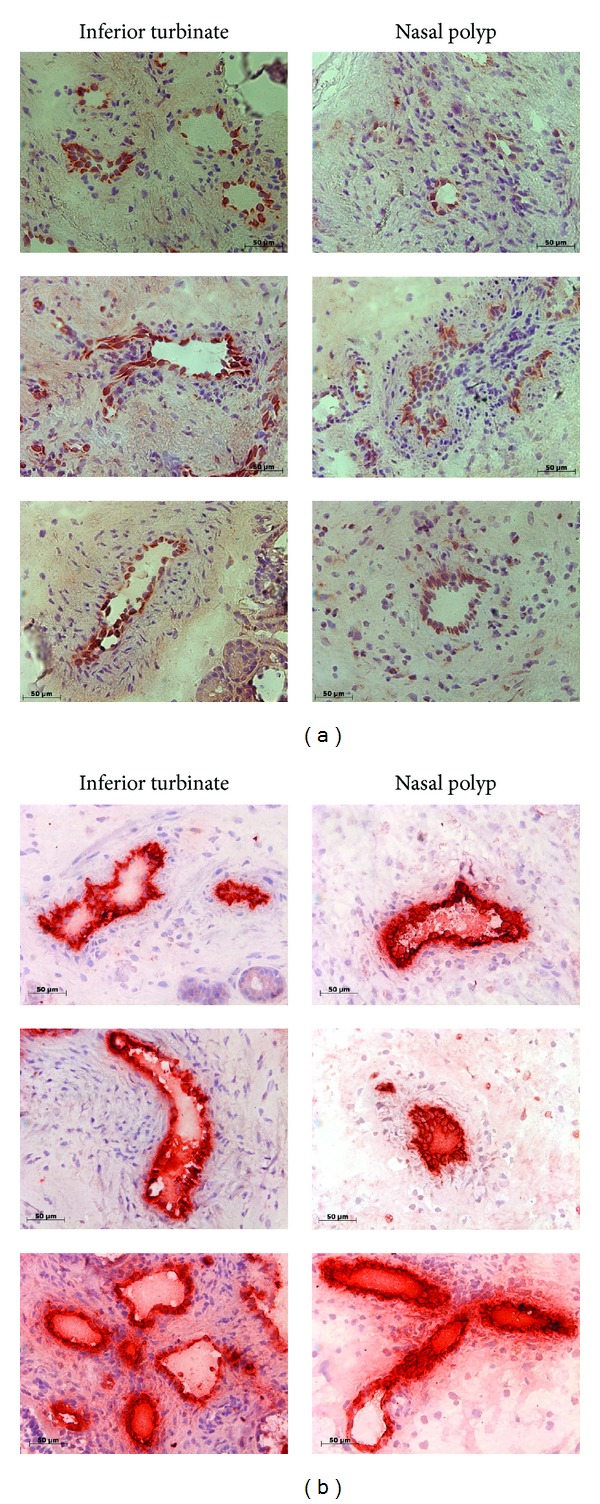
(a): Expression and localization of E-selectin in inferior turbinates and nasal polyps of chronic rhinosinusitis patients (*n* = 10). Immunohistochemical staining was performed on frozen tissue sections of inferior turbinate (left) and nasal polyp (right). We were able to detect E-selectin positive cells in the endothelium of inferior turbinates and nasal polyps. E-selectin was expressed at high level in the inferior turbinates, whereas in nasal polyps E-selectin was infrequently expressed and always at a low level. (b) Expression and localization of P-selectin in inferior turbinates and nasal polyps of chronic rhinosinusitis patients (*n* = 10). Immunohistochemical staining was performed on frozen tissue sections of inferior turbinate (left) and nasal polyp (right). P-selectin was detected in the endothelium of inferior turbinates and nasal polyps. P-selectin was always expressed at high levels in both inferior turbinates and nasal polyps.

E- and P-selectin positive cells were detected in the endothelium of nasal polyps and inferior turbinates (*n* = 10). E-selectin was expressed at high levels in the inferior turbinates (2+ or 3+), whereas in nasal polyps E-selectin was irregularly expressed and always at a low level (negative or 1+, Figure 4(a)). P-selectin was expressed at high levels (3+) in the endothelium of all observed vessels in both inferior turbinates and nasal polyps (Figure 4(b)). Additional staining showed increased expression of VCAM1 in sections of nasal polyps compared to the inferior turbinates and similar expression of ICAM1 (data not shown).

### 3.4. Eosinophil and Neutrophil Counts

**Figure 5:**
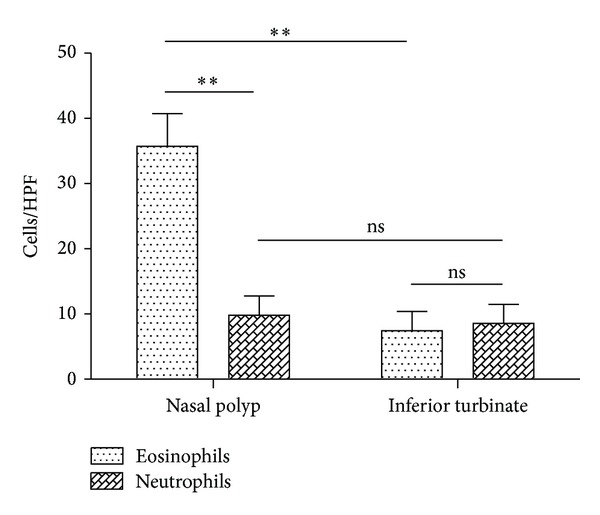
Eosinophils and neutrophils counts in nasal polyps and inferior turbinates (*n* = 8). Eosinophil counts were significantly higher in nasal polyps compared to inferior turbinates (*P* = 0.007). Neutrophil counts did not significantly differ between nasal polyps and inferior turbinates. Noticeable is that there was a significant difference between eosinophil and neutrophil counts in nasal polyps (*P* = 0.0014) and balanced counts in inferior turbinates.

Examination of stained sections revealed that eosinophil counts were significantly higher in nasal polyps compared to inferior turbinates (35.7 ± 12.24 versus 7.43 ± 7.8 eosinophils/HPF, *P* = 0.007). Neutrophil counts did not significantly differ between nasal polyps and inferior turbinates (9.8 ± 7.84 versus 8.55 ± 7.18 neutrophils/HPF). Strikingly there was a significant difference between eosinophil and neutrophil counts in nasal polyps (35.7 ± 12.24 versus 9.8 ± 7.84 cells/HPF, *P* = 0.0014) and balanced counts in inferior turbinates (7.43 ± 7.8 versus 8.55 ± 7.18 cells/HPF) (Figure 5).

## 4. Discussion

Despite several hypotheses that have been put forward regarding the pathogenesis of chronic rhinosinusitis with nasal polyps (CRSwNP), there is still need for a more detailed understanding of the basis for inflammation, including the mechanisms responsible for recruitment and activation of leukocytes in nasal polyps. Inflammation in CRSwNP and CRSsNP is characterized by distinct inflammation patterns, showing a relatively Th1 biased inflammation pattern for CRSsNP in both Caucasians and Asians, while CRSwNP portrays Th2-biased inflammation in Caucasians and is Th1/Th17 biased in Asians [2].

The recruitment of circulating blood leukocytes to sites of inflammation involves the expression of adhesion molecules by endothelial cells [6]. Several studies suggest a role of adhesion molecules for the pathogenesis of allergic, chronic, and acute inflammatory diseases [14–17] as well as tumor growth and metastasis [18, 19]. In both bronchial biopsies and serum of asthmatic patients, increased expression levels of E-selectin, ICAM1, and VCAM1 have been observed [20–23], suggesting a role for the pathophysiology of inflammatory airway diseases [24]. In order to understand better the mechanisms responsible for recruitment and activation of leukocytes in nasal polyps, we studied the expression patterns of adhesion molecules in nasal polyp and associated inferior turbinate tissue.

Vascular cell-adhesion molecule 1 (VCAM1) conveys the adhesion of eosinophils, basophils, monocytes, and lymphocytes. E-selectin and ICAM1 are known for a selective recruitment of eosinophils and neutrophils, whereas VCAM1 plays a favored role for eosinophil extravasation in chronic inflammatory conditions [2, 25, 26]. Regarding the different expression of VCAM1 between microarray and qPCR analysis, we think that the reason for this is that qPCR analysis is more sensitive than microarray analysis. Additionally, we analyzed more patients during qPCR analysis which led to a relativization of VCAM1 expression on gene level. Others reported an increased expression of VCAM1 in nasal polyps [26, 27] and we also could see this on protein level. ICAM1 plays a dominant role in allergic rhinitis and asthma. It initiates and modulates different intercellular signaling events and cellular functions and is highly related to the proinflammatory infiltrate [28, 29]. Nonetheless, ICAM1 is not specific for the lymphocyte response mainly of the Th2 type. In line with our results, Cavallari et al. and Jahnsen et al. observed no difference in the expression of ICAM1 between nasal polyps and inferior turbinates, but its role in CRSwNP has not been clarified despite its high levels of expression [26, 30, 31]. Apart from VCAM1, P-selectin has been suggested primarily to promote the recruitment of eosinophils in nasal polyp tissues. In line with our results, others have reported that P-selectin is well expressed in nasal polyp tissue [27, 32]. But this upregulation could not explain the selective recruitment in nasal polyps because P-selectin is also able to bind neutrophils. However, when we put these results together, an upregulation of P-selectin and VCAM1 would conceivably explain such selective recruitment of eosinophils. Furthermore, a downregulation of E-selectin could also play an important role in this selectivity, as it was seen in Figure 5.

In simple terms, E-selectin can be regarded as a relative counterpart of VCAM-1 and P-selectin, which preferentially promotes the recruitment of neutrophils [33]. Both E-selectin and P-selectin are able to support binding of both neutrophils and eosinophils, but E-selectin is most efficient at raising the affinity of CD18 integrins that support neutrophil deceleration and trafficking to sites of acute inflammation [33]. Symon et al. showed that only 29% of total blood vessels were stained positive for E-selectin [32]. In fact we found an irregular and low expression of E-selectin in nasal polyps. Ural et al. [34] concluded that it is not entirely justifiable that E-selectin has a role in the pathogenesis of intranasal polyposis.

However, this downregulation of E-selectin may be a crucial factor in the pathogenesis of nasal polyps, because E-selectin optimizes the mechanics and kinetics for the recognition of multiple ligands. The bond strength of E-selectin is more durable than those of P-selectin, because the membrane tethered with the substrate transmits force to the bonds with E-selectin ligands under shear forces of blood flow [33]. Most nasal polyp tissues of western population are characterized by selective recruitment of eosinophils and mononuclear cells, but not neutrophils. In the case of E-selectin a blocking antibody inhibited the early recruitment of neutrophils in a primate model, but not the influx of eosinophils [25, 32]. The role of E-selectin in immune surveillance may be to amplify the sensitivity to activation with chemokines at sites of vascular inflammation and to locally activate neutrophil arrest and migratory function [33]. Neutrophils were suggested to play a significant role in the resolution of inflammation as well as for the pathology of the chronic inflammatory state [35].

Following the concept of a united airways disease, we would have expected increased expression levels of E-selectin in nasal polyps, as it has been reported for bronchial biopsies and serum of asthmatic patients [20–23]. Our results show lower expression of E-selectin in the endothelium of nasal polyps, which has also been shown for blood vessels of human squamous cell carcinomas of the skin, in which the downregulation of vascular E-selectin was suggested to help malignant cells to evade the immune response [36].

Consistently with our results, recent studies have demonstrated that CRSwNP in Caucasians is a Th2 disorder [2]. It has been shown that TNF*α* induces the E-selectin gene transcription and consequently increases the recruitment of neutrophils [37]. Furthermore IL-1*β* is also able to induce the E-selectin gene expression [38], while IL-4 downregulates its expression [37].

However, an imbalance between Th1 (IL-1*β*, IL-2, IFN*γ*, and TNF*α*) and Th2 responses (IL-4, IL-5, IL-10, and IL-13) led to a chronic inflammatory answer and to establish the final balance between Th1 and Th2 may be essential for the enhancement or protection of disease [39, 40]. E-selectin is responsible for the cellular influx of neutrophils and is downregulated by the Th2 cytokine IL-4. In line with the downregulation of E-selectin, we demonstrated a selective recruitment of eosinophils in nasal polyps (Figure 5). In contrast there was a final balance between eosinophils and neutrophils in inferior turbinates. Thus, a downregulation of E-selectin may come along with a significantly reduced influx of neutrophils in nasal polyps.

## 5. Conclusion

On the basis of our results, we suggest that an upregulation of E-selectin and the associated influx of neutrophils may be essential to reestablish the immune balance in Caucasian nasal polyps, which are mostly dominated by eosinophils. In order to understand the pathogenesis and development of Caucasian nasal polyps, the complex network of leukocyte migration needs further investigation in future studies to elucidate underlying inflammatory patters.

## Figures and Tables

**Table 1:** TaqMan Gene expression assays used for qPCR.

Gene symbol	Assay ID	GeneBank	Length of amplicon
*β*-Actin	Hs99999903_m1	NM_001101.3	171 bp
E-selectin	Hs00950401_m1	NM_000450.2	104 bp
P-selectin	Hs00174583_m1	NM_003005.3	63 bp
PSGL1	Hs00380945_m1	NM_003006.3	68 bp
ICAM1	Hs99999152_m1	NM_000201.2	99 bp
VCAM1	Hs00365486_m1	NM_080682.2, NM_001199834.1, NM_001078.3	122 bp
CD31	Hs00169777_m1	NM_000442.4	65 bp

**Table 2:** Comparison of CD31 and *β*-actin normalized qPCR data.

Gene	Relative expression	
	Normalized to CD31	Normalized to *β*-actin
E-selectin	0.22-fold	0.31-fold
P-selectin	1.82-fold	1.97-fold
PSGL1	1.29-fold	1.34-fold
ICAM1	0.93-fold	1.28-fold
VCAM1	1.56-fold	1.08-fold
